# Reducing Passive Drug Diffusion from Electrophoretic Drug Delivery Devices through Co‐Ion Engineering

**DOI:** 10.1002/advs.202003995

**Published:** 2021-04-10

**Authors:** Shao‐Tuan Chen, Megan N. Renny, Liliana C. Tomé, Jorge L. Olmedo‐Martínez, Esther Udabe, Elise P. W. Jenkins, David Mecerreyes, George G. Malliaras, Robert R. McLeod, Christopher M. Proctor

**Affiliations:** ^1^ Electrical Engineering Division Department of Engineering University of Cambridge Cambridge CB3 0FA UK; ^2^ Materials Science and Engineering Program University of Colorado Boulder CO 80309 USA; ^3^ POLYMAT, University of the Basque Country UPV/EHU Avenida Tolosa 72 Donostia‐San Sebastian 20018 Gipuzkoa, Spain; ^4^ Ikerbasque Basque Foundation for Science Bilbao 48013 Spain; ^5^ Department of Electrical, Computer & Energy Engineering University of Colorado Boulder CO 80309 USA

**Keywords:** bioelectronics, device optimization, electrophoretic transport, targeted drug delivery

## Abstract

Implantable electrophoretic drug delivery devices have shown promise for applications ranging from treating pathologies such as epilepsy and cancer to regulating plant physiology. Upon applying a voltage, the devices electrophoretically transport charged drug molecules across an ion‐conducting membrane out to the local implanted area. This solvent‐flow‐free “dry” delivery enables controlled drug release with minimal pressure increase at the outlet. However, a major challenge these devices face is limiting drug leakage in their idle state. Here, a method of reducing passive drug leakage through the choice of the drug co‐ion is presented. By switching acetylcholine's associated co‐ion from chloride to carboxylate co‐ions as well as sulfopropyl acrylate‐based polyanions, steady‐state drug leakage rate is reduced up to sevenfold with minimal effect on the active drug delivery rate. Numerical simulations further illustrate the potential of this method and offer guidance for new material systems to suppress passive drug leakage in electrophoretic drug delivery devices.

## Graphical abstract

Drug leakage is a safety concern for long‐term implantable electrophoretic drug delivery devices. In this work, co‐ion engineering is proposed as a clinically viable method to drastically reduce drug leakage rate, without any sacrifices on the device active performance. The co‐ion engineering method is drug‐independent and can be readily applied to existing electrophoretic drug delivery devices.

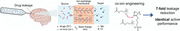

## 1 Introduction

Electrophoretic drug delivery devices can be implanted directly into the targeted treatment site, bypassing physiological obstacles such as the blood‐brain barrier^[^ ^1^ ^]^ and can achieve higher efficacy while delivering lower dosage compared to systemic administrations.^[^ ^2^ ^]^ In contrast to other implantable drug delivery methods such as convection‐enhanced delivery devices,^[^ ^3^ ^]^ electrophoretic drug delivery does not increase local pressure by injecting solvent, reducing risks of backflow,^[^ ^4^ ^]^ and issues with long‐term device reliability.^[^ ^5^ ^]^ Instead of solvent flow, electrophoretic devices use an applied electric field to push drugs across an ion‐conducting membrane which in turn allows for precise control of the rate of drug delivery and, ideally, a high ratio of drug flow between active and idle states (i.e., ON/OFF ratio). Electrophoretic drug delivery devices encompass a growing family of devices that include different architectures such as the microfluidic ion pump^[^ ^6^ ^]^ and the capillary organic electronic ion pump^[^ ^7^ ^]^ which have previously shown promise for addressing a wide range of physiological conditions from epilepsy to stress in plants.^[^ ^8^, ^9^, ^10^, ^11^, ^12^, ^13^ ^]^

Despite this success, limiting drug leakage when the device is idle remains a hurdle to long‐term implantation. The flow of drug from the device when it is intended to be OFF could cause side effects or buildup of drug tolerances; not to mention drug leakage reduces the lifetime of the drug source reservoir thereby requiring more frequent refills. Reducing drug leakage is ever more important considering recent advancements in the field that have relied on ever thinner membranes to reduce power requirements and enhance drug delivery rates.^[^ ^6^, ^10^, ^14^ ^]^ Previous attempts to limit diffusive drug leakage for electrophoretic drug delivery devices include increasing membrane resistance,^[^ ^15^ ^]^ using a bipolar membrane in a diode configuration rather than a single cation or anion exchange membrane,^[^ ^16^ ^]^ increasing the concentration ratio between fixed charge concentration in the membrane to source reservoir and applying a reverse “retaining” potential during the idle state.^[^ ^17^ ^]^ While many of these efforts have proven effective, these solutions increase the energy required to deliver drugs to a therapeutic level thereby reducing the device power efficiency and/or drug delivery rate. Here, we use a combination of experimental work and computational modeling to demonstrate the universal applicability of a new method of reducing drug leakage in electrophoretic drug delivery devices with minimal impact on other performance metrics: changing the drug co‐ion in the source reservoir.

The concept of ion pairing has been utilized in drug designs to alter the hydrophilic/hydrophobic balance of drug molecules.^[^ ^18^, ^19^, ^20^, ^21^ ^]^ Pairing hydrophilic drug molecules with hydrophobic co‐ions/peptides can increase the permeability of drug/co‐ion pair into cell membranes,^[^ ^22^, ^23^ ^]^ enhance transdermal penetration depth,^[^ ^24^ ^]^ or increase the drug solubility.^[^ ^25^ ^]^ Ion‐pairing can tailor the pharmacokinetics of drugs to different applications without modifying its chemical structure. As a result, this approach increases the chance for regulatory approval and making it a highly attractive method for developing new medications.^[^ ^26^ ^]^ For electrophoretic drug delivery devices where drug leakage poses a major challenge for long‐term implantation safety and device reliability, the potential of pairing charged drug molecules with different co‐ions to reduce the leakage rate has not been previously explored.

In this work, a drug leakage suppression method, termed co‐ion engineering, is reported. The theoretical framework describing the relationship between co‐ion diffusion coefficient and drug leakage rate from an electrophoretic drug delivery device is presented and validated with experimental work. Further numerical simulations demonstrate the applicability and consistent leakage reduction capability of co‐ion engineering to a variety of drugs commonly used in electrophoretic drug delivery devices. This study shows co‐ion engineering can achieve significant drug leakage suppression without sacrificing active device performance or requiring additional operational power. We posit this method can be readily used with a variety of drugs across existing electrophoretic drug delivery devices.

## 2 Results

### 2.1 Drug Transport Mechanisms in an Electrophoretic Drug Delivery Device

**Figure 1:**
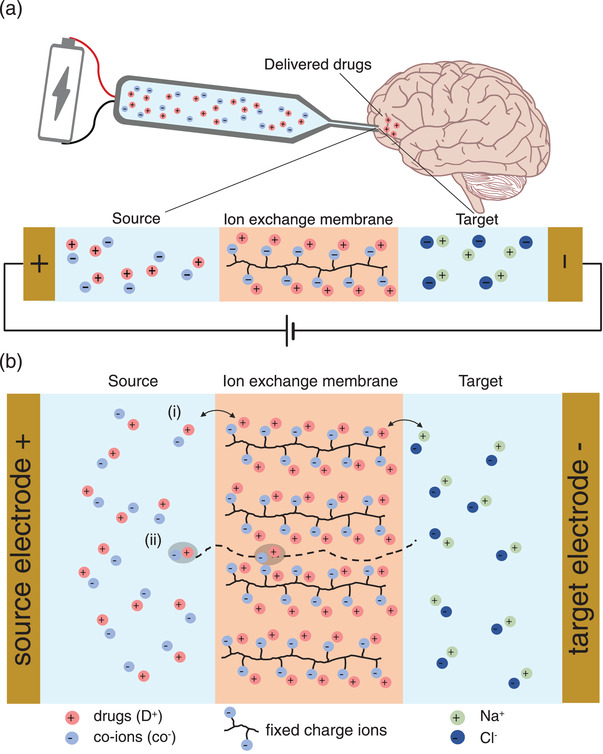
Working principle of an electrophoretic drug delivery device. a) Schematic showing different components of an electrophoretic drug delivery device in relation to the implanted area for in vivo applications (not to scale). b) Schematic of mass transport in an electrophoretic drug delivery device. Two drug permeation pathways, (i) Counter‐ion exchange (IE) and (ii) Associated ion diffusion (AID) exist during both active and idle states.

An electrophoretic drug delivery device consists of a source drug reservoir with a source electrode, an ion exchange membrane (IEM) at the point of drug delivery and a target electrode in contact with the target site (e.g., tissue area immediately external to the implant) (Figure 1a). When the device is in operation, an electric potential is applied between the source and target electrode, transporting the charged drug molecules from the source across the ion exchange membrane and into the target site. The charge of the drug and IEM are chosen deliberately such that the IEM only allows migration of the drug while selectively blocking transport of oppositely charged ions from target back to the source. When the device is idle and no external potential is applied, drug transport across the IEM is significantly reduced however drug leakage across the IEM may still occur due to the concentration gradients between the source, IEM, and target.

Drug leakage in an electrophoretic drug delivery device can be understood by analyzing the mass transport mechanisms from the drug source reservoir through the IEM into the target site (**Figure** **1b**). The IEM is a polymeric membrane containing fixed charge groups^[^ ^27^ ^]^ and in recent years IEMs have been the most commonly reported type of ion‐conducting membrane used in electrophoretic devices due to their charge selectivity. The IEM separates the drug reservoir, or source, from the target. In both active and idle states, the ionic flux *J_i_* for species *i* through an IEM of an electrophoretic drug delivery device is described by the 1D Nernst‐Planck equation along the *x*‐direction as:^[^ ^28^ ^]^

(1) $$J_{i}=\;-D_{i}\left(\frac{da_{i}}{dx}+\frac{z_{i}FC_{i}}{RT}\frac{d\varphi }{dx}\right)$$

where *D_i_* is diffusion coefficient, *a_i_* is the activity (*a_i_ = γ_i_C_i_*), *C_i_* is concentration, *z_i_* is charge number, *F* is Faraday's constant, *R* is the gas constant, *T* is temperature, and φ is the applied electric potential. In this study, we take *γ_i_* = 1 and so *a_i_ = C_i_*. In a standard electrophoretic drug delivery device, the high drug activity gradient at the IEM‐target interface drives drug out of the membrane and into the target where it is typically metabolized or transported away via convection.^[^ ^29^, ^30^ ^]^ The continued loss of drug creates a concentration gradient $\frac{dC}{dx}$ in the membrane, and this concentration gradient is due to an irreversible thermodynamic process, regardless of the presence of an external voltage. Reducing this concentration gradient has been the focus of previous reports aiming to limit drug leakage.^[^ ^15^, ^31^ ^]^ However, these approaches introduce additional power consumption due to increased ionic resistance. Likewise, reducing the drug concentration in the source reservoir simultaneously reduces the reservoir lifetime as well as the drug delivery rate.^[^ ^32^ ^]^

In this work, we focus on altering the *D_i_* in Equation 1 with the aim of reducing drug leakage without a concurrent effect on power consumption or active drug transport. We hypothesized this may be possible as there are two major pathways for concentration‐driven diffusion of charged molecules across an IEM:^[^ ^33^ ^]^ ion exchange^[^ ^34^ ^]^ (IE) and associated ion diffusion^[^ ^35^ ^]^ (AID). In IE, a source counter‐ion, which is an ion of opposite charge to fixed ions, exchanges with a counter‐ion that was electrostatically coupled to a fixed‐ion in the IEM.^[^ ^35^ ^]^ After diffusing across the membrane through IE from fixed‐ion to fixed‐ion, these counter‐ions can once again undergo IE with available counter‐ions in the target. The second mechanism, AID, is when a counter‐ion dissolves into the membrane as part of a “charge neutral pair” with a co‐ion. The ions in this pair are considered associated ions and their transport parameters are coupled. In the absence of an applied electric field, AID is understood to be the primary diffusion mechanism at steady‐state for an IEM separating electrolytes of different concentrations.^[^ ^27^, ^36^ ^]^ Assuming nonideal thermodynamic factors such as the mismatch in activity coefficients between species in source and target are small,^[^ ^37^ ^]^ the overall effective coupled diffusion coefficient *D*_s_ of the charge‐neutral pair has previously been derived by accounting for electrostatic interactions (counter‐ion condensation), tortuosity effect and concentration difference in the IEM.^[^ ^38^, ^39^ ^]^ The coupled diffusion coefficient is :

(2) $$D_{s}=\frac{D_{\mathrm{drug}}^{m}D_{\mathrm{co}}^{m}\left(C_{\mathrm{drug}}^{m}+C_{\mathrm{co}}^{m}\right)}{D_{\mathrm{drug}}^{m}C_{\mathrm{drug}}^{m}+D_{\mathrm{co}}^{m}C_{\mathrm{co}}^{m}}$$

where $D_{\mathrm{drug}}^{m},D_{\mathrm{co}}^{m},\;\;C_{\mathrm{drug}}^{m},C_{\mathrm{co}}^{m}$ with superscript *m* represent the diffusion coefficient and concentration values of drug and co‐ions in the IEM.

Considering Equations 1 and 2, it is observed that at steady‐state the overall mass transfer of drug due to concentration‐driven diffusion in the absence of an applied field is dependent on the diffusion coefficient of the associated co‐ions. In theory, one could therefore suppress the drug diffusing from the source reservoir into the target by designing a system with a small co‐ion diffusion coefficient. Critically, one would not expect that the co‐ion diffusion coefficient would affect drug transport in an applied electric field as such transport primarily occurs via IE and is independent of *D*_co_.^[^ ^40^ ^]^ With this theoretical basis in mind, we conducted a series of experiments to measure the effect of co‐ions on drug leakage.

### 2.2 Drug Leakage Profile When Paired with Co‐Ions of Similar Molecular Weight

Acetylcholine (ACh), a drug commonly used in organic electronic ion pumps, was paired with a series of carboxylate co‐ions with increasing alkyl chain length, specifically butyrate (But), hexanoate (Hex), and octanoate (Oct) (see Scheme S1 in the Supporting Information). These new acetylcholine salts were prepared by using typical protocols for ionic liquid synthesis (see Supporting Information). Choosing carboxylate as co‐ions allow us to adjust the molecular weight by increasing the carbon chain length while avoiding other potential interactions between the drug and co‐ions. The goal is to establish the relationship of co‐ion diffusion coefficient *D*_co_ on drug leakage rate from the IEM of an electrophoretic drug delivery device. It is worth remarking that all of salts used in this study are within the family of bioactive ionic liquids based on natural compounds which may have applications in other drug delivery technologies, where the toxicity of such salts is reported to be negligible for concentration lower than molar ranges.^[^ ^41^, ^42^, ^43^ ^]^

**Figure 2:**
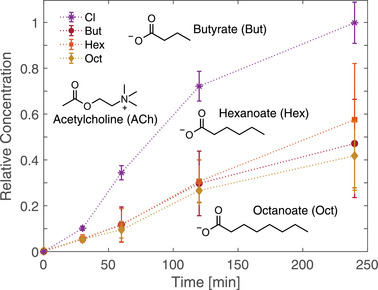
Time‐dependent drug diffusion profile when paired with carboxylate co‐ions. Acetylcholine leakage profile when the source solution was an aqueous electrolyte of acetylcholine:carboxylate salt of variable carbon‐chain lengths. Inset: molecular structure of acetylcholine, butyrate, hexanoate, and octanoate. Relative concentration is calculated by normalizing against ACh Cl concentration at 240 min.

**Figure** **2** shows the relative ACh concentration measured in the target as a function of time when paired with a series of carboxylate co‐ions using a custom‐made testing cell with a standard source‐IEM‐target layout (Figure S1, Supporting Information), with acetylcholine chloride as comparison. Measurements were conducted using a polystyrene sulfonate (PSS) based IEM with no externally applied field (see Experimental section). For all timepoints the average concentration of diffused ACh was highest for ACh Cl with a roughly 55% reduction in drug leakage observed for the largest co‐ion pairing (ACh Oct). However, differences in measured ACh concentration between the carboxylate co‐ions were within measurement error (95% confidence interval for at least three samples). The results can be understood in terms of *D*_co_ by employing the modified Stokes‐Einstein equation, a power‐law relationship between diffusion coefficient *D* and molecular weight *M* $(D\;\propto M^{-\frac{1}{\alpha }},\;\alpha \;=\;2.56-3).$ ^[^ ^44^, ^45^ ^]^ Using this analysis as a first approximation of the relative changes in *D*_co_, we estimate a roughly 40% decrease in *D*_co_ for Oct compared to *D*_co_ for Cl and a no more than 18% relative reduction in *D*_co_ when comparing Oct and But co‐ions. The results in Figure 2 thus suggest that modest changes in *D*_co_ can lead to modest changes in drug leakage, and in order to achieve meaningful drug leakage reduction, the change in *D*_co_ needs to be significant.

### 2.3 Drug Leakage Profile When paired with Co‐Ions with Orders of Magnitude Difference in Molecular Weight

To further explore the potential effects of D_co_ on drug leakage, we coupled ACh to polymeric co‐ions and performed diffusion experiments. The co‐ion of choice, sulfopropyl acrylate (SPA), was paired with ACh to form the monomer acetylcholine 3‐sulfopropyl acrylate (ACh SPA). SPA monomers and polymers are a family of biocompatible compounds suitable to be used in implantable drug delivery applications. SPA polymers have previously been used in biomedical applications including gastro retentive drug delivery systems,^[^ ^46^, ^47^ ^]^ superporous hydrogel for drug delivery,^[^ ^48^, ^49^ ^]^ polymer coatings for medical devices,^[^ ^50^, ^51^ ^]^ and implantable concentration gradient driven power sources for bioelectronics.^[^ ^52^ ^]^ By synthesizing polyanion forms of the monomer ACh SPA through free radical polymerization and inverse emulsion (see Supporting Information for further synthetic procedures), we prepared poly(sulfopropyl acrylate) polyanions with ACh as counter‐cations and molecular weight on the order of 85 000 g mol^‐1^ for low MW poly(SPA ACh) and >1000 000 g mol^‐1^ for high MW poly(SPA ACh), respectively. Following the same calculations based on the modified Stokes‐Einstein equation, we estimate the *D*_co_ for low MW and high MW poly(SPA ACh) to be approximately 10–12% and 4–5% to that of the ACh SPA monomer respectively. It should be noted this estimate is applicable to diffusion in a solvent and, though the IEM is hydrated, it may still significantly underestimate the reduction in diffusion coefficient for polymeric co‐ions within the IEM.

**Figure 3:**
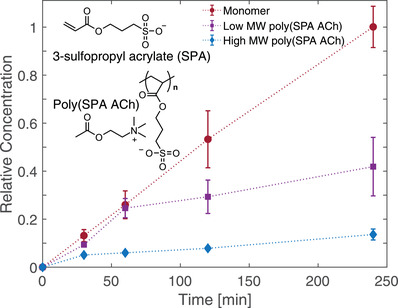
Time‐dependent drug diffusion profile when paired with SPA monomer and polymers. Acetylcholine time‐dependent leakage profile when paired with a series of acetylcholine:poly(sulfopropyl acrylate)s. Inset: molecular structure of sulfopropyl acrylate (SPA) monomer and polymer. Relative concentration is calculated by normalizing against ACh SPA monomer concentration at 240 min.

**Figure** **3** shows the relative ACh concentration in the target as a function of time when paired with the ACh SPA monomer, low MW poly(SPA ACh), and high MW poly(SPA ACh) with data points normalized to the ACh concentration with the SPA monomer at 240 min. Within 60 min, all three samples are observed to approach a constant steady‐state rate of acetylcholine leakage. Comparing drug solutions with the three different co‐ions, a significant reduction in the steady‐state drug leakage rates is observed when ACh was paired with the polymers as co‐ions. For the low MW SPA polymer, the steady‐state leakage rate was reduced fourfold, while further switching to high MW SPA polymer resulted in nearly sevenfold reduction in drug leakage compared to the monomeric form of SPA. These findings illustrate the powerful role of the drug co‐ion in drug leakage across an IEM in an electrophoretic drug delivery device. A sevenfold reduction could lead to an equivalent extension of the time between refilling drug solutions. Moreover, drugs typically have a strong dose dependence, so even a twofold reduction in leakage rate could mark the difference between safe operation and undesirable effects.^[^ ^53^ ^]^

### 2.4 Relation between Drug Leakage Profile and Diffusion Coefficient via Computational Modeling

**Figure 4:**
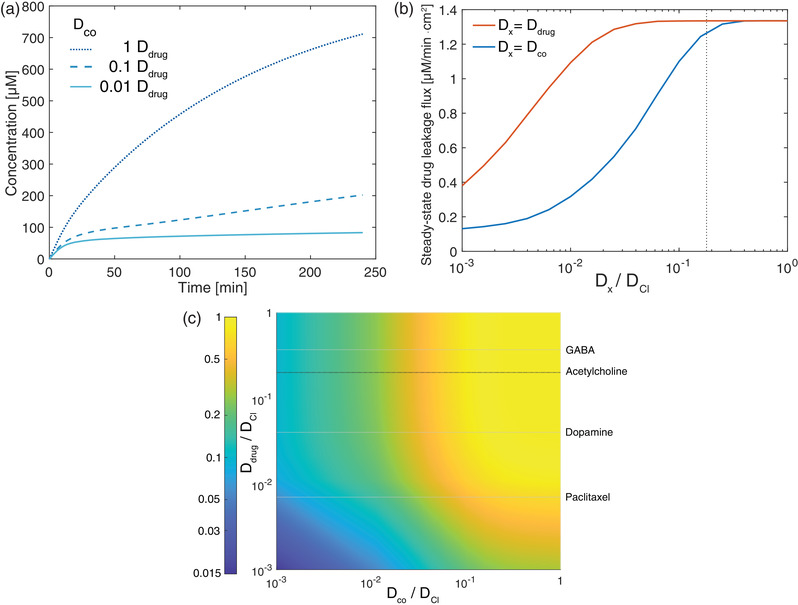
Numerical simulation results for drug leakage dependence on co‐ion diffusion coefficient. a) Time‐dependent drug leakage profile for different values of *D*_co_ with *D*_drug_ fixed as *D*_ACh_. b) Simulation of steady‐state drug leakage flux as a function of co‐ion (blue line) and drug (orange line) diffusion coefficients. Dotted line represents the diffusion coefficient for ACh. c) Contour plot of steady‐state drug leakage as functions of both drug and co‐ion diffusion coefficients. Color bar indicates log scale of normalized drug leak rate. Dotted lines represent diffusion coefficient for GABA, ACh, dopamine, and paclitaxel.

To better understand the role of *D*_co_ in particular on drug leakage and to explore whether the change in *D*_co_ can explain the findings presented in Figures 2 and 3, we conducted numerical simulations using our recently reported computational model developed for electrophoretic drug delivery devices.^[^ ^32^ ^]^ The 1D model mirrors the geometry of a microfluidic ion pump^[^ ^6^ ^]^ with blocking electrode boundary conditions. The temporal behavior of drug leakage was explored with three sets of time‐dependent numerical simulations where *D*_co_ between each run was reduced by an order of magnitude (**Figure** **4a**) with the *D*_drug_ and initial *D*_co_ both equal to *D*_ACh_. As in the experimental work, the simulated IEM was loaded with drug at the start of each simulation, causing a higher transient leakage rate as a result of the high initial electrochemical potential gradient. The time‐dependent drug leakage profiles demonstrate similar characteristics as shown in Figure 3. The steady‐state drug leakage fluxes were found to be 1.1, 0.32, and 0.13 [µM (min* cm^2^)^‐1^] from high to low *D*_co_ solutions by extracting the slope of these three curves from 150 min onwards. These results indicate that the steady‐state drug leakage rate can be reduced by approximately three times and eight times by reducing *D*_co_ by one and two orders of magnitude respectively. That these simulation results are on par with the experimental findings in Figure 3c supports the notion that the change in *D*_co_ is the primary reason for the observed reduction in diffusion when comparing ACh with SPA monomer and polymer co‐ions.

Having observed the importance of *D*_co_ in steady‐state drug leakage, numerical simulations were further employed to explore a wider range of diffusion coefficient considering both *D*_co_ and *D*_drug_. Figure 4b shows the steady‐state drug leakage rate for two systems where *D*_co_ and *D*_drug_ are alternatively fixed at *D*_ACh_ while the other is varied over three orders of magnitude relative to *D*_Cl_. The leakage rate is observed to depend strongly on *D*_co_ (blue line) in the range of roughly 10^−2^ to 10^−1^ *D*_Cl_. A pronounced saturation in drug leakage rate is observed as *D*_co_ decreases to 10^−3^ *D*_Cl_ and likewise as *D*_co_ increases above *D*_ACh_ (noted by the dotted line). In contrast, the steady‐state drug leakage rate is remarkably independent of *D*_drug_ across nearly two orders of magnitude with a significant decrease starting only when as *D*_drug_ falls below roughly 10^−2^ *D*_Cl_ (Figure 4b, orange line). Extending the simulations to consider simultaneous changes in *D*_co_ and *D*_drug_ indicates a similar dependence between *D*_co_ and steady‐state drug leakage for *D*_drug_ ranging from 1 to 10^−2^ *D*_Cl_ with an even more pronounced reduction in drug leakage as *D*_drug_ approaches 10^−3^ *D*_Cl_ (Figure 4c). For reference, dashed lines indicate values for *D*_drug_ of commonly used compounds GABA, ACh, dopamine, and paclitaxel, a notably larger charged drug that may be possible to deliver in the future with further advancements in the development of IEMs for electrophoresis.^[^ ^12^, ^14^, ^54^ ^]^ The contour plot shows the remarkable contrast in the effects of *D*_drug_ and *D*_co_ on drug leakage rate persist across the spectrum of relevant diffusion coefficient values; whereas a decreasing *D*_drug_ by a factor of 100 may have a negligible effect on steady‐state drug leakage rate, the equivalent reduction in *D*_co_ could reduce the leakage rate fivefold. As illustrative examples, for GABA, ACh, dopamine, and paclitaxel, the steady‐state drug leakage rate when paired the same co‐ion are nearly identical for *D*_co_ ranging from 1 to 10^−2^ *D*_Cl_. These findings can be understood in terms of Equation 2. Due to Donnan exclusion, $C_{\mathrm{co}}^{m}\ll C_{\mathrm{drug}}^{m}$, and therefore a change in *D*_Drug_ would be effectively cancelled out whereas a reduction in *D*_co_ would directly reduce the effective coupled diffusion coefficient. It is only when *D*_drug_ is reduced to a factor similar to the ratio of $C_{\mathrm{co}}^{m}/C_{\mathrm{drug}}^{m}$ that the drug leakage rate is noticeably reduced as well.

From a clinical perspective, the significance of these results translates to the wide applicability and consistent drug leakage suppression ability for the co‐ion engineering method. Co‐ions with higher molecular weight can be widely applied to a variety of drugs to reduce the drug leakage rate, despite the fact that *D*_drug_ for these drugs vary over two orders of magnitude. Furthermore, since the steady‐state drug leakage rate is nearly identical in this regime, the proposed co‐ion engineering method is drug‐independent. The biocompatible SPA co‐ion system shown here can therefore be further used by a range of drugs while achieving the same effect of drug leakage suppression, eliminating the need for future researchers to synthesize different co‐ions tailored to drugs used in different applications.

### 2.5 Active Performance for Electrophoretic Drug Delivery Devices When ACh is Paired with Different Co‐Ions

**Figure 5:**
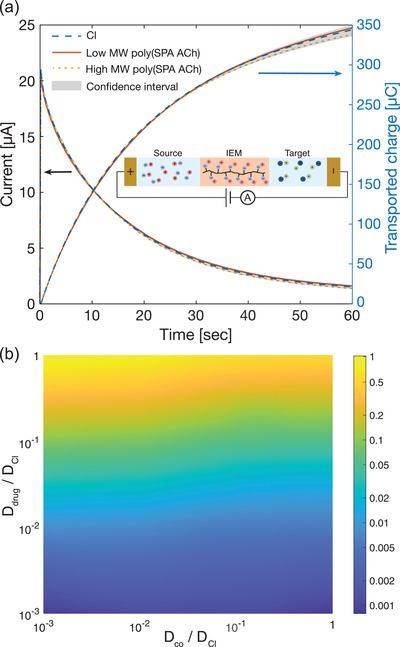
Device performance during active state. a) Current and charge versus time data for ACh transport by varying co‐ions from Cl to high MW SPA polymer (averaged results where *n* = 4 for each co‐ion sample). Inset: Circuit connecting scheme for applying constant voltage and measuring the electric current passing through the driving circuit. b) Contour plot of active drug transport as functions of both drug and co‐ion diffusion coefficient. Color bar indicates log scale of normalized drug delivery rate.

Finally, we investigated how the choice of co‐ion affects drug transport with an applied voltage. A microfluidic ion pump device with a PSS‐based membrane^[^ ^6^ ^]^ was prepared with three different 10 × 10^‐3^ m source solutions of ACh combined with Cl, low MW SPA polymer, and high MW SPA polymer co‐ions. A 0.5 V potential was applied between the source and target electrodes with phosphate‐buffered saline in the target well (see Experimental section). The 0.5 V operation voltage was chosen such that it is both within the safety limit for in vivo applications^[^ ^6^, ^10^ ^]^ and does not introduce additional drug diffusion.^[^ ^32^, ^55^ ^]^ Notably, the measured current and transported charge were found to be nearly identical between the three co‐ion samples (**Figure** **5a**). Simulations from our computational model of the microfluidic ion pump further illustrate that active drug transport is largely independent of co‐ion diffusion coefficient (Figure 5b). Likewise, the simulation shows electrophoretic drug delivery depends strongly on the *D*_Drug_ with an approximately linear correlation consistent with previously reported experiments.^[^ ^17^, ^29^, ^56^ ^]^ The results in Figure 5b follow from the established theory of electrophoresis in IEMs—most notably the Nernst‐Plank relation and associated expressions for the conductivity of an IEM. Also, according to Marcus theory for ion pairing,^[^ ^40^ ^]^ with the presence of an external electric field, the charged drug is carried across the membrane faster and so the drug salt in the source will dissociate to compensate this unequal loss of drug counter‐ion and associated pairs. As a result, IE would be the dominant transport mechanism (see Supporting Information).

These results demonstrate that the drug co‐ion can be chosen to minimize drug leakage without affecting the active drug delivery rate. The findings also suggest that a change in binding affinity between ACh and SPA poly‐SPA polymer compared to ACh and Cl is unlikely to be a significant contributor to the decreased transport in either the on or off state. (A method for estimating the ionic strength for a drug with different co‐ions can be found in Supporting Information). By the same reasoning, Figure 5a indicates that the reduction of drug leakage in the poly (ACh poly‐SPA ACh) system cannot be explained by the potential entrapment of ACh molecules in the poly‐SPA polymer matrix.

## 3 Discussion and Conclusion

All together the results presented here indicate that drug leakage in IEM based electrophoretic delivery devices can be significantly reduced by coupling drugs to slow‐moving co‐ions. Critically, the use of slower co‐ions does not come at the cost of active drug delivery rates nor operational power requirements. Though we focus on anionic co‐ions here, the same approach should equally apply to cationic co‐ions. As this approach concerns only a change to the drug solution rather than the device geometry, it can be readily integrated into the variety of reported electrophoretic device architectures which to date have relied almost exclusively on the use of drugs with small co‐ions such as Cl.^[^ ^8^, ^9^, ^12^, ^13^, ^30^, ^56^ ^]^ We posit that the simulation results in Figure 4c may prove particularly useful in guiding future design of new drug co‐ion systems with drug leakage rates tailored for the application.

To summarize, we demonstrate, drug leakage in IEM based electrophoretic drug delivery devices can be suppressed without affecting the active device performance by changing the associated co‐ion in the drug solution. By way of example, we show that the steady‐state leakage rate of acetylcholine can be reduced up to sevenfold by changing its associated co‐ion from chloride or carboxylates of increasing alkyl chain length (C4 to C8) to poly(sulfopropyl acrylate). Active drug delivery experiments show that the choice of co‐ions in the drug solution does not affect the amount of drug delivered with an applied voltage. Comparing experimental results with numerical simulations, we further demonstrate that the strategy presented in this paper is compatible with a range of drugs commonly used in electrophoretic drug delivery devices. The simulation results can be used to guide the design of future drug co‐ion systems for optimal drug leakage reduction. The method presented here can be readily applied to other electrophoretic drug delivery device architectures thereby extending device lifetime and enabling safe operation for long‐term implantations.

## 4 Experimental Section

##### Membrane Preparation

The membrane was made of over‐oxidized polyethyldioxythiophene doped with polystyrene sulfonate (PEDOT:PSS). The PEDOT:PSS was mixed as dodecylbenzyl sulfonic acid [0.1% w/w] from Sigma‐Aldrich (Spain), ethylene glycol [5 % w/w] from Fisher Scientific (Spain), and Clevios PH 1000 supplied by Heraus (Spain) [94.9% w/w]. To this, 3‐glycidmethoxypropyl silane from Sigma‐Aldrich was added [1 % w/w]. After 3 min of sonication, 250 µL of this solution was drop cast on Whatman Cyclopore Polycarbonate (Sigma‐Aldrich, Spain), track etched membrane with a nominal pore size of 5 µm. The membrane surface was activated by plasma treatment for 150 s prior to drop casting. After drying overnight, films soaked for 25 s in 3:1 deionized water:Clorox bleach and then rinsed in deionized water. Films were measured to be about 4.3 µm thick.

##### Electrolyte Preparation

The butyric acid (≥99 wt% pure), octanoic acid (≥99 %), ACh chloride (≥99 %), 3‐sulfopropyl acrylate potassium salt (KSPA, 96 %), 2,2′‐azobis(2‐methylpropionamidine) dihydrochloride (AIBA, 97%), Isopal L (>90%), Span 83 (>60%), and sodium metabisulfite (SMB, >99 %) were purchased from Sigma‐Aldrich (Spain). The potassium hydroxide (KOH, ≥85%) was supplied by Fisher Scientific (Spain), while hexanoic acid (≥99 %) and Softanol 90 were provided by Acros Organics (Spain) and Quimidroga (Spain), respectively. All these chemicals were used as received. The solvents were of analytical grade and used without further purification.

##### Diffusion Experiments

The test‐cell assembly with IEM was placed on a hotplate/stirrer, which was set to 25 °C and 60 rpm. A stir bar was placed only on the target side reservoir during tests and glass coverslips were used to cover each side to prevent any evaporation. The source side was filled with 2.5 mL of 10 × 10^‐3^ m ACh electrolyte and the target side was filled with 2.5 mL of phosphate‐buffered saline solution prepared as suggested. At regular times, the target size coverslip was removed only long enough to collect 200 µL of the target side solution. The level of solution on the target side was always above the porthole in the PDMS gasket for the duration of the experiment so that the mass transfer area of the membrane did not change. Each sample was assumed to be well mixed. Before switching to a new ACh:anion pair, the membrane was flushed on both sides with deionized water at least five times. All samples were stored in a 4 °C refrigerator until assayed for ACh concentration.

##### Colorimetric Assay

A colorimetric assay of ACh was used to quantify the amount of ACh that had diffused into the target via an ACh assay kit [MBS169077] provided by MyBioSource (San Diego, CA, USA). Samples taken from cool storage were centrifuged to ensure accurate sample concentrations. 50 µL of sample were mixed with 100 µL of the 1× assay buffer (the provided 10× assay buffer diluted to a 10^th^ its concentration) to ensure all samples readings would fall inside the detection range of the kit. As suggested by the kit, 50 µL of these diluted samples were added to a 96‐well plate (cellstar) in duplicate which sat on ice to ensure the integrity of the samples. Reaction mix was prepared as suggested: choline oxidase (25 µL), HRP (10 µL), colorimetric probe (100 µL), and acetylcholinesterase (20 µL) mixed and diluted with 1× assay buffer to a total volume of 5 mL in a multichannel pipette reservoir. Standards were also prepared as suggested: 10 µL of the provided acetylcholine standard was mixed into 490 µL of the 1× assay buffer to obtain the upper bound standard concentration (200 µmol). Half the volume of the upper bound was combined with an equal volume 1× assay buffer to obtain 100 µmol standards, and this process was repeated to obtain successive standards of half the previous concentration down to 0.78 µmol. Pure 1× assay buffer was used to provide the control blank. 50 µL of standards were added to the well plate in quadruplicate. A multichannel micropipette was used to distribute 50 µL of the well‐mixed reaction mix to each well, such that each well containing a sample or standard held 100 µL, and the plate was incubated at room temperature for 1 h away from light.

Finally, the plate was read using a Spectramax ID3 Multi‐Mode Microplate Reader (Molecular Devices, San Jose, CA, USA). Again, as suggested, the plate reader was set to read at the 540 nm wavelength. Precision of the assay kit can be examined by referring to Figure S3 (Supporting Information), the acetylcholine standard curve which is expected to be a linear relationship between absorbance and ACh concentration. The signal‐to‐noise ratio of the colorimetric assay varies from 16 to 25 dB depending on the ACh concentration of the measured sample.

##### Active Pumping Experiments

Electrophoretic drug delivery devices were prepared according to a previous report.^[^ ^6^ ^]^ The devices were loaded with solution consisting of 10 × 10^‐3^ m ACh paired with either Cl, low MW SPA polymer, or high MW SPA polymer with a phosphate buffered saline solution in the target site. The applied potential was controlled using an Autolab N‐series Potentiostat (Metrohm Autolab, The Netherlands) with a custom‐designed software interface (Autolab NOVA). The electrophoretic drug delivery device was operated in constant voltage mode, where a DC voltage of 0.5 V was applied between the source and target electrodes integrated on the device,^[^ ^6^ ^]^ with the circuit connecting scheme shown in the inset of Figure 5a. The resulting current passing through the driving circuit was recorded by the potentiostat with a sampling rate of 20 Hz. Subsequently, following the previously established protocol,^[^ ^8^, ^17^, ^56^ ^]^ transported charge was obtained by integrating the current versus time data. The current measurement has a signal‐to‐noise ratio >85 dB when the measured current is above 1 µA.

##### Computational Modeling

The governing equations of the 1D model were solved by finite element method using COMSOL 5.4 software (COMSOL Multiphysics, V5.4, USA). Initial conditions were set where the source reservoir contains both counter‐ions and co‐ions with a concentration of 10 × 10^‐3^ m, and the target reservoir contains 160 × 10^‐3^ m of NaCl to mimic physiological conditions. Fixed charge concentration in the IEM was set as 0.5 m, resulting an IEM with pumping efficiency *η* of 0.95. For boundary conditions, the electrodes of the electrophoretic drug delivery device were assumed to be perfectly polarizable, with no ionic flux entering or leaving from the electrode surface. No restriction of influx and efflux between electrolyte–IEM interface was imposed on any charged particles considered in the study. For both diffusion and active pumping simulations, the final drug concentration in the target reservoir was obtained by considering both the influx from IEM to the target interface and the integral of drug concentration along the entire length of the target reservoir. Adaptive mesh density scheme was used, where the length of the mesh elements in the bulk of reservoirs was set to be 10^−10^ m, and 10^−12^ m at the electrode–reservoir and reservoir–IEM interfaces to ensure convergence. Diffusion coefficients for charged particles considered in the model are as follow: *D*_Na_+ = 1.33 × 10^−9^ m^2^ s^−1^, *D*_Cl_− = 2.03 × 10^−9^ m^2^ s^−1^, with both *D*_drug_ and *D*_co_ varying from 2.03 × 10^−9^ to 2.03 × 10^−12^ m^2^ s^−1^ with a 0.1 reduction factor for the diffusion coefficient of charged species in the IEM.^[^ ^16^, ^27^ ^]^

## Conflict of Interest

The authors declare no conflict of interest.

## Supporting information

Supporting Information

## Data Availability

Research data are not shared.
