# Supplementary material for: Reducing Passive Drug Diffusion from Electrophoretic Drug Delivery Devices through Co‐Ion Engineering
Source: Adv Sci (Weinh). 2021 Apr 10;8(12):2003995. doi: 10.1002/advs.202003995 (PMC8224430; doi:10.1002/advs.202003995)
Supplement: Supplementary file 1 — Supporting Information [file ADVS-8-2003995-s001.pdf]

© 2021 Wiley-VCH GmbH

ADVANCED SCIENCE
Open Access

Supporting Information

for *Adv. Sci.*, DOI: 10.1002/advs.202003995

Reducing Passive Drug Diffusion from Electrophoretic Drug Delivery Devices through Co-ion Engineering

Shao-Tuan Chen‡, Megan N. Renny‡, Liliana C. Tomé, Jorge L. Olmedo-Martínez, Esther Udabe, Elise P.W. Jenkins, David Mecerreyes, George G. Malliaras, Robert R. McLeod* and Christopher M. Proctor**

Supporting Information

Reducing passive drug diffusion from electrophoretic drug delivery devices through co-ion engineering

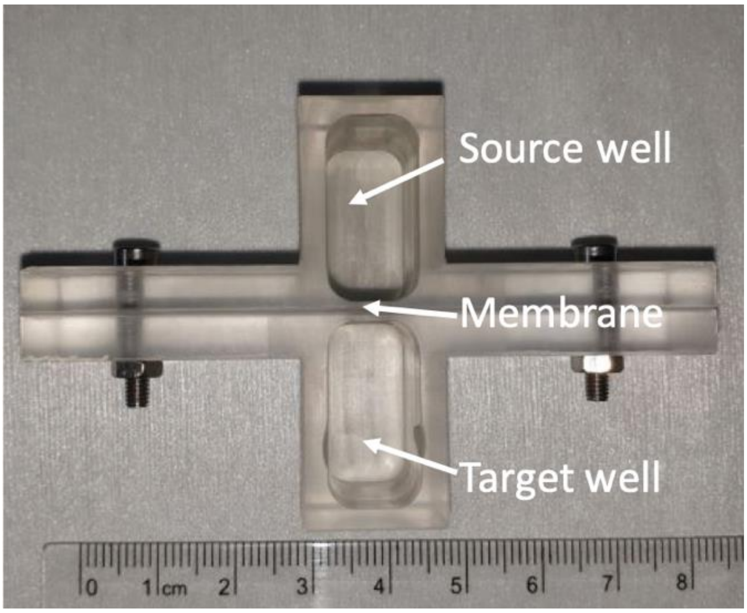

Figure S1. *Custom-made test cell for membrane characterization, employed for diffusion experiment in this study.*

Synthetic Procedures

Synthesis of ACh Carboxylate Ionic Liquids

ACh-based ionic liquids combining carboxylate anions with variable carbon-chain lengths, including ACh butyrate (ACh But), ACh hexanoate (ACh Hex) and ACh octanoate (ACh Oct), were synthesized *via* a two-step ion exchange method at room temperature (Scheme 1 **A**). In a typical procedure, potassium hydroxide (0.95 mol) was first dissolved in a minimum amount of water and the desired carboxylic acid (1 mol) was added dropwise. After water removal by rotary evaporation, the obtained precipitate was washed with excess of diethyl ether and filtrated. The formed potassium carboxylate salt (1 mol) and ACh chloride (1 mol) were separately dissolved in a minimum amount of ethanol and then mixed by stirring for 1 hour. The mixture was kept in the freezer overnight to complete the precipitation of KCl,

which was then filtered off. After solvent removal by rotary evaporation, the obtained ACh carboxylate ionic liquids were dried under high vacuum. The chemical structures were confirmed by NMR analysis.

ACh butyrate: ^1H NMR (400 MHz, D_2O): δ/ppm = 4.01 (m, 2H, OC**H₂**); 3.47 (t, 2H, NC**H₂**); 3.16 (s, 9H, N(C**H₃**)₃); 2.23 (t, 2H, C**H₂**COO); 1.96 (s, 3H, C**H₃**COO); 1.54 (m, 2H, CH₃C**H₂**); 0.87 (t, 3H, C**H₃**CH₂). ^{13}C NMR (101 MHz, D_2O): δ/ppm = 181.42 (CH₂**C**OO); 179.07 (CH₃**C**OO); 67.66 (t, N**C**H₂); 55.82 (O**C**H₂); 54.09 (t, N(**C**H₃)₃); 37.61 (**C**H₂COO); 21.99 (**C**H₃COO); 18.80 (CH₃**C**H₂); 13.21 (**C**H₃CH₂).

ACh hexanoate: ^1H NMR (400 MHz, D_2O): δ/ppm = 4.02 (t, 2H, OC**H₂**); 3.48 (t, 2H, NC**H₂**); 3.16 (s, 9H, N(C**H₃**)₃); 2.25 (t, 2H, C**H₂**COO); 1.97 (s, 3H, C**H₃**COO); 1.54 (m, 2H, C**H₂**CH₂COO); 1.25 (m, 4H, CH₃(C**H₂**)₂); 0.83 (t, 3H, C**H₃**CH₂). ^{13}C NMR (101 MHz, D_2O): δ/ppm = 181.32 (CH₂**C**OO); 178.82 (CH₃**C**OO); 67.34 (t, N**C**H₂); 55.53 (O**C**H₂); 53.80 (t, N(**C**H₃)₃); 35.32 (**C**H₂COO); 30.66 (**C**H₂CH₂COO); 24.62 (CH₃CH₂**C**H₂); 21.68 (d, CH₃**C**H₂, **C**H₃COO); 13.17 (**C**H₃CH₂).

ACh octanoate: ^1H NMR (400 MHz, D_2O): δ/ppm = 4.02 (m, 2H, OC**H₂**); 3.48 (m, 2H, NC**H₂**); 3.17 (s, 9H, N(C**H₃**)₃); 2.14 (t, 2H, C**H₂**COO); 1.96 (s, 3H, C**H₃**COO); 1.54 (m, 2H, C**H₂**CH₂COO); 1.25 (m, 8H, CH₃(C**H₂**)₄); 0.82 (t, 3H, C**H₃**CH₂). ^{13}C NMR (101 MHz, D_2O): δ/ppm = 184.01 (CH₂**C**OO); 173.19 (CH₃**C**OO); 64.51 (t, N**C**H₂); 58.29 (O**C**H₂); 53.72 (t, N(**C**H₃)₃); 37.41 (**C**H₂COO); 31.02 (**C**H₂CH₂COO); 28.66 (CH₃(CH₂)₃**C**H₂); 28.23 (CH₃(CH₂)₂**C**H₂); 25.81 (CH₃CH₂**C**H₂), 21.98 (CH₃**C**H₂), 20.14 (**C**H₃COO), 13.37 (**C**H₃CH₂).

Synthesis of poly(sulfopropyl acrylate ACh)

First, the monomer ACh 3-sulfopropyl acrylate (ACh SPA) was prepared (Scheme 1 **B**). ACh chloride (5 g, 27.5 mmol) and 3-sulfopropyl acrylate potassium salt (6.4 g) were previously dissolved in a minimum amount of methanol and then mixed under vigorous stirring at room temperature for 1 hour. A catalytic amount of 4-methoxyphenol was added as an inhibitor and the methanol was stripped off under reduced pressure at temperature ≤ 40 ºC. Afterwards, ethanol was added in order to precipitate the KCl, which is a by-product of the anion exchange reaction. The mixture was kept in the freezer overnight to complete the precipitation of KCl. The precipitate was removed by filtration and the excess of ethanol was

gently evaporated. Finally, the resulting white solid monomer was thoroughly dried under high vacuum at room temperature for 2 days.

ACh 3-sulfopropyl acrylate:^{1}H NMR (400 MHz, D_2O): δ/ppm = 6.47–5.77 (m, 3H, C**H_2**=C**H**); 4.45 (m, 2H, OC**H_2**$CH_2N(CH_3)_3$); 4.20 (m, 2H, OC**H_2**$(CH_2)_2SO_3$); 3.63 (m, 2H, NC**H_2**); 3.11 (s, 9H, N(C**H_3**$)_3$); 2.92 (m, 2H,C**H_2**SO_3); 2.12–1.89 (m, 5H, C**H_3**COO, C**H_2**CH_2SO_3).^{13}C NMR (101 MHz, D_2O): δ/ppm =173.14 (CH_3**C**OO); 168.58 (CH_2=CH**C**OO); 132.22 (**C**H_2=CH); 127.50 (CH_2=**C**H); 66.44 (t, N**C**H_2); 63.52 (O**C**$H_2(CH_2)_2SO_3$); 58.26 (O**C**$H_2CH_2N(CH_3)_3$); 53.68 (t, N(**C**$H_3)_3$); 47.66 (**C**H_2SO_3); 23.60 (**C**$H_2CH_2SO_3$); 20.12 (**C**H_3COO).

The polyanion, poly(sulfopropyl acrylate ACh), i.e. poly(SPA ACh), was synthesized by conventional free radical polymerization in water (Scheme 1 **B**). In a typical procedure, 2 g of the prepared ACh SPA monomer were dissolved in 18 g of MiliQ water to have a 10 wt% solid concentration. The monomer solution was purged with dry nitrogen for 20 min before being putted into an oil bath at 70 ºC. The AIBA initiator (1 wt% to the monomer) was dissolved in water, purged with nitrogen and added dropwise to the monomer solution at 70 ºC. The mixture was then allowed to react for 4 h with constant and vigorous stirring. After polymerization, the excess of water was removed by rotary evaporation. The resulting product was then dried under high vacuum at 60 ºC for 24 h to remove traces of water and the polyanion was obtained as a white solid.

In order to obtain a very high molecular weight polyanion, the poly(SPA ACh) was also synthesized by inverse emulsion polymerization (Scheme 1 **C**). The monomer ACh SPA (1.5 g, 4.31 mmol) was dissolved in 0.6 g of water. Isopal L (1.1 g) was used as organic solvent. Span 83 (0.1 g) and Solftanol 90 (0.15 g) were selected as emulsifiers and sodium metabisulfite (SMB) (0.1 g) as initiator. The polymerization reaction was performed at 70 ºC for 2 h, under argon atmosphere. The resulting polymer was then precipitated with cold diethyl ether and thoroughly dried under high vacuum at 60 ºC for 24 h.

The chemical structures of the synthesized polymers were confirmed by ^{1}H NMR. The success of the polymerization reaction can be easily seen from the ^{1}H NMR spectra shown in

Figure S2. The signals associated with the acrylic protons between 5.8 and 6.4 ppm of the ACh SPA monomer disappeared after both polymerization reactions, revealing that complete conversion of the monomer was achieved. In addition, the characteristic chemical shifts arising from the 3-sulfopropyl acrylate polyanion backbone can be clearly observed together with the proton signals associated with the ACh counter-cation. The molar mass of the synthesized polymers was analyzed by SEC/MALS/RI. The equipment was composed by a LC20 pump (Shimadzu) coupled to a miniDawn Treos multiangle (3 angles) light scattering laser and an OptilabT- Rex differential refractometer (all from Wyatt Technology Corp., USA). Separation was carried out using three columns in series (Ultrahydrogel 120, 250, and 2000 with pore sizes of 120, 250, and 2000 A, respectively, Waters, Barcelona, Spain). The analyses were carried out at 35°C, using a 0.1M LiCl/1.5x10^{-5}M NaN3 solution in a mixture of water/acetonitrile (4:1 v/v) at a flow rate of 0.6 ml/min. The refractive index increment was dn/dc= 0.1 ml/g.

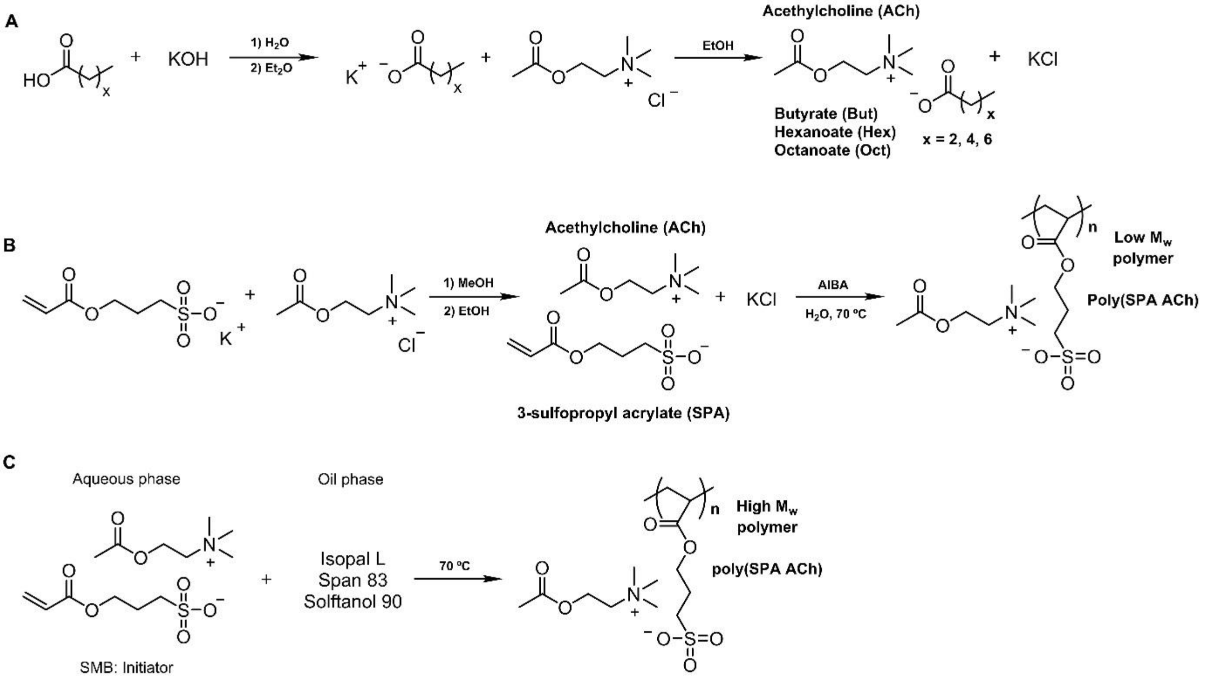

***Scheme 1**: Synthetic pathways for (A) ACh-based ionic liquids combining carboxylate anions, (B) ACh SPA monomer and respective free radical polymerization and (C) inverse emulsion polymerization to form poly(SPA ACh).*

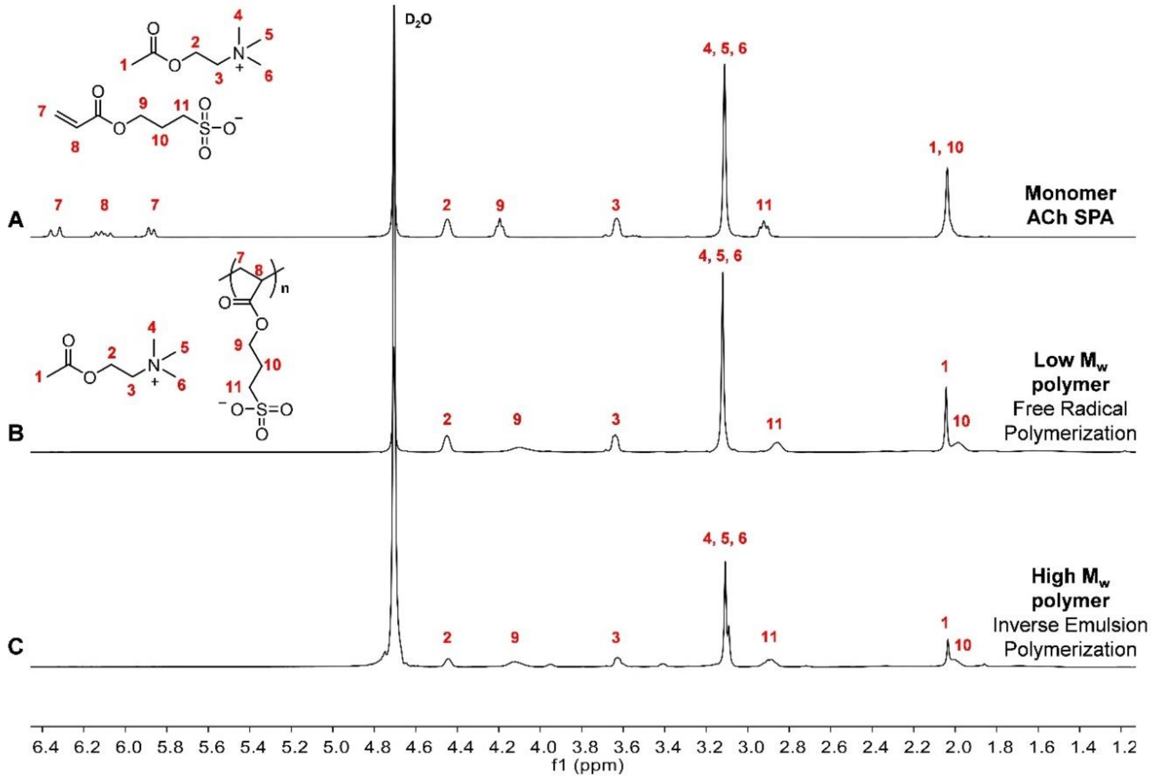

Figure S2. *^{1}H – NMR spectra of (A) ACh 3-sulfopropyl acrylate monomer, and respective polyanions obtained by (B) free radical polymerization and (C) inverse emulsion polymerization.*

Acetylcholine Standard Curves

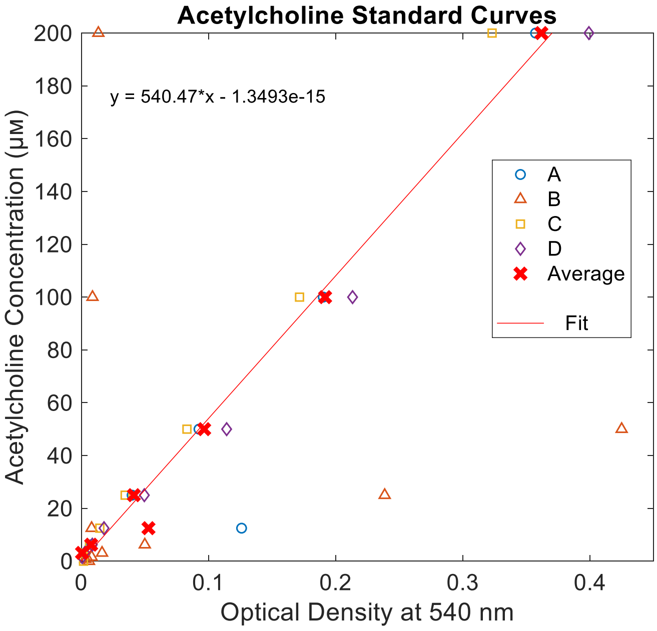

Figure S3. *Standard curves used to determine Acetylcholine concentration in samples. Each data series is the same standard solutions placed at 4 different locations in the plate. Series B*

was not included in the average. The equation is a linear fit to the average where readings using this conversion have an error of 11.2%

Ionic Strength (μ) Consideration:

The drug may spontaneously associate or dissociate with the co-ion in the electrolyte. Such reaction, characterized by changes in hydration sphere necessary to accommodate the different forms, can be written for this process:

ACh:co • xH2O ↔ ACh+ • yH2O + co^- • zH2O. (S1)

The left side of the reaction details the associated form of the drug and the right side of the reaction details the dissociated form. The ratio of dissociated to nominal drug concentrations at equilibrium can be described by the ionic strength, μ.

Marcus Theory for ion pairing states that the energy cost to isolate a multivalent anion from cations is higher than isolating a monovalent anion from the cation. Therefore, in an electrophoretic drug delivery device, the portion of associated ion pairs between ACh and a multivalent high MW SPA polyanion is higher than that of a monovalent chloride system. The ionic strength μ between the drug and co-ion in the electrolyte would dictate the portion between IE and AID pathway.

During active drug pumping, IE is the dominant mass transfer mechanism not only because it is faster due to a larger driving force but because the ratio of dissociated drug remains constant, as dictated by μ, while the total concentration of associated drug diminishes. In the absence of an external electric field, the portion of drug diffusion through IE versus AID for a given drug co-ion system may be proportional to the ionic strength μ of that given system.

The total amount of drug diffusion F_{total} through the two mechanisms at steady-state can therefore be written as:

$$F_{total} = \left[\mu D_{drug} \frac{\mu S}{l} + (1-\mu) D_s \frac{(1-\mu)S}{l}\right], \text{(S2)}$$

where S is the solubility coefficient, D_{drug} and D_s is the drug diffusion coefficient and coupled diffusion coefficient between drug and co-ion in the membrane, and l is the membrane thickness.

Membrane conductivity derivation:

The measurement results shown in Figure 3(a) can be understood by relating the measured current to the total ionic flux transported described Nernst-Planck equation.

During drug transport, the total ionic current I with i species is given as:

$$\mathrm{I} = \mathrm{F} \sum_{\mathrm{i}} \mathrm{z_i J_i}, \text{(S3)}$$

Plug Equations S3 into the Nernst-Planck equation (Equation 1 in main text), we obtain:

$$\mathrm{I} = -\frac{\mathrm{F}^2}{\mathrm{RT}} \sum_{\mathrm{i}} \mathrm{z_i^2\, C_i^m D_i^m} \frac{\mathrm{d}\psi}{\mathrm{dx}}. \quad \text{(S4)}$$

The membrane conductivity for an ion exchange membrane is defined as:

$$\mathrm{K} = \frac{-\mathrm{I}}{\frac{\mathrm{d}\psi}{\mathrm{dx}}}, \quad \text{(S5)}$$

plug in Equations S5 back to Equation S4, the membrane conductivity K can be expressed as a function of the valency, concentration and diffusion coefficient of all the species as:

$$\mathrm{K} = \frac{\mathrm{F}^2}{\mathrm{RT}} \left(\mathrm{z_{drug}^2 D_{drug} C_{drug} + z_{Co}^2 D_{Co} C_{Co} + z_{Na}^2 D_{Na} C_{Na} + z_{Cl}^2 D_{Cl} C_{Cl}}\right). \quad \text{(S6)}$$

Under this framework, changing D_{co} has the least amount of effect on the overall membrane conductivity, since C_{co} is lowest than the rest of the three ions. As a result, further decreasing

D_{co} it would not contribute to significant change in membrane conductivity and the amount of transported charge with the same applied voltage.
